# mSphere of Influence: Virology in the noise—how cell-to-cell variability impacts viral infection outcomes

**DOI:** 10.1128/msphere.00438-23

**Published:** 2023-09-25

**Authors:** Nir Drayman

**Affiliations:** 1 The Department of Molecular Biology and Biochemistry, The Center for Virus Research and The Center for Complex Biological Systems, The University of California, Irvine, Irvine, California, USA; University of Michigan, Ann Arbor, Michigan, USA

**Keywords:** single cell, virus, herpes, HSV-1

## Abstract

Nir Drayman works at the intersection of virology and single-cell biology, studying how cellular heterogeneity shapes the outcome of viral infections (and specifically that of HSV-1). In this mSphere of Influence article, he reflects on how two papers, “Remote activation of host cell DNA synthesis in uninfected cells signaled by infected cells in advance of virus transmission” (N. Schmidt, T. Hennig, R. A. Serwa, M. Marchetti, and P. O’Hare, J Virol 89:11107–11115, 2015, https://doi.org/10.1128/jvi.01950-15) and “Extreme heterogeneity of influenza virus infection in single cells” (A. B. Russell, C. Trapnell, and J. D. Bloom, Elife 7:e32303, 2018, https://doi.org/10.7554/eLife.32303), impacted his research by trail blazing the analysis of viral infections in single cells, as well as by illuminating what is yet left to discover.

## COMMENTARY

Virologists have always been interested in understating viral infection at its “atomic” unit—that of a single cell. Early investigations of bacteriophages (1, 2) and animal viruses (3 – 5) revealed substantial heterogeneity in progeny production among individually infected cells, leading to the recognition that not all infected cells are equal. I imagine these initial observations both fascinated and frustrated the first generation of virologists, as the technology to study individual cells during infection would take several more decades to mature.

Two main technological breakthroughs—quantitative fluorescence microscopy and single-cell RNA sequencing (sc-RNAseq; separated by about 20 years)—now allow virologists to finally try and answer the question raised by Delbrück in 1945—what are the cell-to-cell differences that control viral infection outcome?

In the first paper, “Remote activation of host cell DNA synthesis in uninfected cells signaled by infected cells in advance of virus transmission” (6), Schmidt et al. explore the spatial organization of DNA replication during multiple rounds of Herpes Simplex virus 1 (HSV-1) infection. HSV-1 belongs to the alpha herpesviruses family and is a large double-stranded DNA virus that replicates in the cell nucleus. As such, HSV-1 competes with host DNA for access to replication resources, such as nucleotides and host enzymes. Prior to this work, it has been well established that HSV-1 blocks host DNA synthesis while promoting viral DNA synthesis in infected cells. Viral DNA synthesis is known to have striking spatial features, where host DNA is marginalized (pushed to the outer rim of the nucleus), while viral DNA is concentrated in distinct sub-nuclear structures known as replication compartments.

Here, Schmidt et al. use bioorthogonal chemistry to label and image both host and viral DNA synthesis. This approach relies on the addition of the nucleotide analog ethynyl deoxycytidine (EdC) to the cells. EdC incorporates into newly synthesized DNA and can then be fluorescently labeled and visualized. Using pulse-labeling with EdC of either uninfected or HSV-1-infected cells, the authors analyze the spatial properties of host and viral DNA synthesis during HSV-1 infection. They confirm the known segregation of host and viral DNA in the initially infected cells but stumble on to a much more striking phenomenon—rings of highly EdC-labeled cells surrounding infected cells. By analyzing infected cells under various conditions, the authors elegantly show that this hyper DNA synthesis in cells adjacent to the infected ones is caused by the initially infected cell and is propagated outward as a plaque begins to form. They conclude their investigation by showing that this reprogramming of adjacent cells is caused by an unknown factor secreted from HSV-1-infected cells.

Honestly, what’s not to love about this paper? It takes an extremely well-studied process in virology (host DNA replication shutoff) and completely turns it around simply by looking at the behavior of individually infected cells under the microscope. This and other work from the O’Hare lab serve as constant reminders that simply imaging individually infected cells can reveal hidden layers of complexity in virus-host interactions and open new avenues of exploration.

This manuscript also highly impacted the way I think about viral manipulation of the host cell-cycle. While it is well established that most viruses manipulate the host cell-cycle, this is often attributed to a non-physiological artifact in cell culture experiments. However, I find it hard to believe that such an intricate mechanism as the one described by Schmidt et al., involving a secreted factor that drives nearby cells to hyper replicate their DNA, is merely a cell culture artifact. Rather, I take the view that manipulation of the host cell-cycle is likely to turn out to be an extremely important process *in vivo*.

In the second paper, “Extreme heterogeneity of influenza virus infection in single cells” (7), Russell et al. describe one of the first uses of single-cell RNA sequencing to analyze cell-to-cell variability during influenza A virus (IAV) infection. By leveraging scRNAseq, the authors study IAV infection at a very low multiplicity of infection (MOI), such that most cells are uninfected, and most infected cells replicate a single viral genome. This is in contrast to most virus-host interaction studies, which use high MOI to limit cell-to-cell variability. There are many insights gained from this study, of which I’d like to emphasize two.

The first point is that virus-infected cells show extreme heterogeneity in the levels of viral gene expression and viral replication. This has since been shown for many viral infections and might even be taken for granted by some of the readers but has been an uphill battle to get into a consensus. Russel et al. made a seminal contribution to this debate by showing that 6–8 hours post IAV infection less than 10% of cells were responsible for over 50% of viral transcripts! In other word, the distribution of viral transcripts across infected cells is highly skewed, with most infected cells having low levels of viral transcripts, while a small subset of cells harbors most of the viral transcripts. Russel et al. also introduced the use of the Gini coefficient, often used in economics to measure income inequality, as a way to quantify cell-to-cell heterogeneity in viral gene expression.

The second point is about the nature of the antiviral response. IAV, like many other viruses, has been reported to activate the type I interferon system upon infection, causing nearby cells to go into a defensive mode. Thus, Russell et al. expected to find many cells expressing the interferon genes, as well as cells expressing interferon-stimulated genes (ISGs). To their surprise, only a single cell in their data set had detectable levels of interferon transcripts, and only a few cells expressed ISGs. This manuscript posed a question which, as a field, we have yet to answer—where does interferon come from during viral infection? My post-doctoral work suggested that, at least in the case of HSV-1, interferon is only produced in cells undergoing abortive infections and only in a small subset of those (8).

Overall, this paper was one of the first to pioneer the use of high throughput, system-level approaches to characterize virus-host interactions at the single-cell level. It was, and remains, an inspiration to me on the proper, rigorous way to ask such questions.

In my newly established lab, we continue to try and answer some of the very basic questions in virology using various single-cell approaches. Examples of such questions are how many viruses are produced by an individually infected cell? What leads cells to undergo abortive infections or to become super producers? It is my hope that the answers to these questions will both make our basic understating of viruses more complete and uncover novel points of intervention that could serve to develop new antiviral therapies.
